# High sensitive troponin T elevation in an oncology patient on treatment with Pembrolizumab

**DOI:** 10.1515/almed-2025-0148

**Published:** 2025-11-25

**Authors:** Alberto Alcalde Morales, Miguel Reyes Milla, Rufino Mondéjar García, Antonio León Justel

**Affiliations:** Laboratory Medicine Department, Hospital Universitario Virgen Macarena, Seville, Spain; Laboratory Medicine Department, Hospital Universitario Nuestra Señora de Valme, Seville, Spain; Laboratory Medicine Department, Hospital Universitario Virgen Macarena, Instituto Biomedicina Sevilla IBIs/CSIC/Universidad de Sevilla/Universidad Loyola Andalucia, Seville, Spain

**Keywords:** immune-mediated toxicity, muscle damage, Pembrolizumab, troponins

## Abstract

**Objectives:**

Troponins are a complex of three proteins that play a key role in the contraction of striated muscles. Currently, cardiac troponins T (cTnT) and I (cTnI) are the best biomarkers for acute myocardial infarction (AMI) diagnostic.

**Case presentation:**

Immune-mediated toxicity due to Pembrolizumab was suspected in a 70-year-old male patient with gastric adenocarcinoma. To confirm this, creatine kinase (CK) and high sensitivity troponin T (hs-cTnT) analysis was carried out, giving values above the reference range. While CK normalized over time, hs-cTnT levels remained high. Before opting to perform an endomyocardial biopsy, high-sensitive troponin I (hs-cTnI) was measured, yielding a normal value. Due to discrepancies in troponin levels, a standard protocol was followed to confirm the presence of analytical interferences, that was negative.

**Conclusions:**

The discrepancy observed between both troponin levels, and the absence of analytical interference, led us to exclude cardiac damage in our patient. So, the most probable cause of elevated cTnT was muscle involvement. This case highlights the importance of confirming troponin results using an alternative assay to rule out limited specificity and emphasizes the need for accessible tools to identify potential analytical interferences.

## Introduction

Troponins are proteins that play a key role in the contraction of striated muscles. By binding to cytoplasmic calcium and subsequently to tropomyosin, troponins expose the actin and myosin binding sites, allowing contraction to occur. Troponins are a complex consisting of three protein subunits: troponin C, troponin T, and troponin I. The first subunit is responsible for binding calcium, the second acts as a link to tropomyosin, and the third inhibits the actin-myosin interaction. Each subunit has different specific isoforms depending on the muscle tissue in which they are located and developmental stage of the individual. Notably, the cardiac troponin C isoform is not exclusive to cardiac tissue but is also found in slow-twitch skeletal muscle fibers [1].

When myocardial cell damage occurs, troponins are released to the bloodstream [2]. Currently, cTnT and cTnI are the best biomarkers for AMI diagnosis, due to their higher sensitivity and specificity compared to previously used markers, such as creatine kinase MB isoenzyme [3], 4].

Despite the clear benefits of these biomarkers in the AMI diagnosis, several aspects should be considered when interpreting them. Troponin elevation is mainly related to myocardial damage, with or without necrosis, so it is not fully specific to AMI and can be increased in other events [4], 5]. Furthermore, several studies have shown troponin elevations from non-cardiac causes [6]. The specificity of cTnT may be decreased in conditions such as renal disease or skeletal muscle damage [7]. Besides this, positive and negative interferences may occur due to the presence of endogenous antibodies in patients [8]. Therefore, it is crucial for clinicians and laboratory managers to be aware of these limitations to provide reliable results that enable accurate diagnosis and appropriate treatment.

## Case presentation

A 70-year-old male patient was attended in the General Oncology Department of the Virgen Macarena University Hospital for the treatment of gastric adenocarcinoma. The following month, he started treatment with Pembrolizumab in monotherapy, a monoclonal antibody directed against programmed cell death protein 1. Soon after starting treatment, the patient experienced severe discomfort and was admitted with asthenia, weakness in hands and upper limbs, and difficulty breathing.

Immune-mediated toxicity due to Pembrolizumab was suspected, causing a syndrome of myositis, myasthenia, and myocarditis. To confirm this, a biochemical study was carried out, analyzing CK and hs-cTnT using the Cobas 8000 system (Roche Diagnostics, Basel, Switzerland; hs-cTnT 99th percentile value, 14 ng/L). Both were elevated, confirming this clinical picture. Upon admission, a CK of 485 U/L was detected, which normalized over time. However, hs-cTnT levels fluctuated (from 1,430 ng/L on admission, with a peak of 2,572 ng/L and discharge levels of 1,338 ng/L) (Table 1).

**Table 1:** Comparison between plasma cTnT and CK levels over time.

Sample	1	2	3	4	5	6	7	8	9	10
CK (50–195 U/L)	485	441	277	121	244	330	287	194	228	264
TnT (0.5–14 ng/L)	1,430	1,240	828	684	1,466	2,572	2,185	1,526	1,338	1,955

The oncologist contacted the laboratory to discuss the case, before opting to perform an endomyocardial biopsy. It was decided to determine hs-cTnI. The sample with a cTnT result of 2,185 ng/L was sent to an external laboratory for cTnI testing. The analysis was performed using the Atellica IM 1600 analyzer (Siemens Healthineers, Tarrytown, NY, USA; hs-cTnI 99th percentile value: 45.2 ng/L), yielding a result of 18.6 ng/L.

Due to discrepancies in troponin levels, a standard protocol was followed to confirm the presence of analytical interferences: a new analysis to confirm the results, serial dilutions in plasma, polyethylene glycol precipitation, heterophile antibody elimination, anti-cardiac muscle antibody determination, and exclusion chromatography were performed.

New samples were requested to confirm the results and to investigate possible interference. These samples were analyzed for hs-cTnT using Roche equipment and for hs-cTnI with the Alinity i platform (Abbott Diagnostics, Abbott Park, IL, USA; hs-cTnI 99th percentile value, 34 ng/L). The results showed again differences between the two troponins (Table 2).

**Table 2:** Comparison between hs-cTnT and hs-cTnI concentrations in serum and plasma.

Sample	hs-cTnT (Roche), ng/L	hs-cTnI (Abbott), ng/L
Serum	1,519	15
Plasma	1,444	14.2

Then, we performed a serial dilution for hs-cTnT analysis in plasma which did not indicate interference (Table 3).

**Table 3:** Preservation of linearity after serial dilutions of the serum.

Dilution	Hs-cTnT (Roche), ng/L
1/2	1,688
1/4	2,028
1/8	2,144
1/16	2,480

To check the presence of immunocomplexes, the sample was precipitated with polyethylene glycol 6000 (PEG-6000). For this, 500 μL of patient serum and 500 μL of PEG-6000 solution were added to a vial. This mixture was centrifuged at 3,500 rpm for 30 min at 5 °C. Later, 500 μL of the antibody-free supernatant, was mixed with 500 μL of physiological serum and the sample was analysed. This process results in the final sample having a ¼ dilution. The addition of PEG-6000 resulted in a plasma recovery of 108.6 %, ruling out the presence of immunocomplexes.

Then, tubes coated with a heterophile antibody blocking reagent (HBR) directed against human heterophile antibodies were used to test for heterophile antibody interference, which block interference by steric hindrance. 500 μL of the patient sample was added to the HBR, mixed and incubated for 1 h at room temperature. The removal of heterophile antibodies showed a plasma recovery of 84.3 %. These results discarded the possible interference from immunoglobulins and heterophile antibodies.

Later, to test the possibility of the existence of an interference by anti-cardiac muscle antibodies we sent the sample to an external laboratory. The result was negative. In addition to this, an extended myositis/myasthenia gravis autoantibodies panel was performed with a negative result.

Finally, to rule out the presence of interfering factors which might result in false positive Elecsys cTnT values, the plasma sample was analysed by Roche Diagnostics in a Collaborative Investigation and Research Lab (Penzberg, Germany) fractioning the sample by size exclusion chromatography (SEC). In a first step, the proteins in the sample were separated according to their molecular weight, covering the full molecular size ranged from 0.2 to 970 kDa. Then, all generated SEC fractions were measured with Elecsys cTnT V2.1 sales lot kits. Reactivity with the TnT assay was mainly found in fractions where free cTnT (∼35 kDa) was expected. No increased reactivity was found in fractions where proteins with molecular weight of IgM (∼970 kDa) or IgG (∼150 kDa) were expected. Hence, the presence of such an interfering factor could be excluded.

## Discussion

The immune checkpoints have adverse events, being myocarditis the most common and fatal cardiovascular toxicity with high mortality but reported in few patients [9]. Myositis and myasthenia gravis can overlap the symptoms of adverse events. The case presented here was not trivial, given that this overlapping syndrome is associated with substantial in-hospital mortality [10].

The discrepancy observed between cTnT and cTnI, together with the absence of antibody interference, led us to rule out cardiac damage in our patient (myocarditis). So, the most probable cause of elevated cTnT was muscle involvement. The laboratory has played a key role in this study, ruling out the possible interferences and making possible the measurement of cTnI, in a rapid fashion to discard cardiac damage.

Another important point in this type of case is the value of CK since it is a biomarker of muscle damage [11]. Bibliographic references [6], 7], 12], 13] compare cTnT levels with CK levels, finding a positive correlation between both biomarkers. As we could observed in our case (table 1), both CK and cTnT increase or decrease in similar way. However, as previously mentioned, normal CK values accompanied very high cTnT concentrations (e.g., the fourth value, where CK is 121 U/L and cTnT is 684 ng/L). This could be due to the higher sensitivity of troponin vs. CK [3].

Although this is not the typical presentation, other cases of troponin discrepancy (elevated cTnT with normal cTnI) have been reported in patients with muscle disease [7]. However, to our knowledge, this is the first report of such markedly elevated cTnT levels resulting from muscle injury in a patient receiving immune checkpoint inhibitors. The high cTnT levels observed could have resulted in erroneously ruling out muscular damage, leading to unnecessary cardiac tests and potentially harming the patient, such as an endomyocardial biopsy.

The exact origin of troponin T elevation in the context of muscle injury is unclear, with disparity of arguments. Some authors have reported mRNA cTnT expression in biopsies of regenerating muscle tissue [12]. Nonetheless, Giannitsis et al. questioned the true origin of this increase since these studies did not analyze whether the mRNA is translated into protein [7]. A study which found the protein in muscle biopsies could not explain how much of local cTnT was responsible for the increase in whole blood [13]. Other authors have suggested another possible origin focused on a cross-reactivity of skeletal troponin T with the antibody used in the assay.

Despite cTnI being historically considered less susceptible to non-ischaemic muscular elevation, its immunoassays are known to be more prone to analytical interferences from endogenous antibodies and macrotroponin [8], which can lead to false results. Therefore, clinicians must maintain a high index of suspicion for non-ischaemic and non-analytical elevations.

This case emphasizes the importance of laboratory confirmation using an alternative troponin assay to rule out limited specificity and highlights the need for readily available tools to investigate potential analytical interferences when biochemical results are inconsistent with the patient’s clinical findings.

## Lessons learned

In laboratories utilizing hs-cTnT for diagnosis, it is crucial to remember the lack of specificity of this parameter in the presence of muscle disease. This means that elevated hs-cTnT levels might not always indicate cardiac issues if the patient has underlying muscle conditions.

For the accurate generation of a laboratory report, it is essential to consider not only the patient’s clinical context but also the analytical process of the samples. When the diagnostic suspicion is not clear, potential analytical interferences that could falsify a parameter’s result must be ruled out.
